# Breaking the Barrier: Understanding Esophageal Ruptures

**DOI:** 10.7759/cureus.58373

**Published:** 2024-04-16

**Authors:** Marielle Jamgochian, Keline Peters, Tejinder Kaur, Chandni Lotwala, Aasim I Chaudhry, Jagtar Sekhon, Zeeshan Khan

**Affiliations:** 1 Family Medicine, Robert Wood Johnson University Hospital, Freehold, USA

**Keywords:** metastatic cancer, thoracic surgery, emergency gastroenterology and endoscopy, palliative care, esophageal rupture

## Abstract

Esophageal rupture, though rare, presents as a critical medical emergency demanding swift recognition and intervention. This condition entails a breach in the integrity of the esophageal wall, leading to leakage of its contents into the mediastinum or surrounding structures. Its etiology often involves a combination of factors, including forceful vomiting, foreign body ingestion, or medical procedures like endoscopy. Timely diagnosis through imaging modalities like CT scans, contrast esophagography, or endoscopy is crucial for prompt management and favorable outcomes. Offering aggressive care in the setting of futile treatment for esophageal perforations raises several ethical, medical, and practical implications. If the prognosis is deemed futile due to factors such as extensive tissue damage, underlying comorbidities, or delayed presentation, aggressive care may only prolong suffering without meaningful improvement in outcomes. Opting for palliative measures in such cases focuses on enhancing the patient's quality of life and providing comfort rather than pursuing futile treatments.

## Introduction

Esophageal rupture, though rare, presents a critical medical emergency requiring swift recognition and intervention. The following case report elaborates on this uncommon condition. This condition entails a breach in the integrity of the esophageal wall, leading to leakage of its contents into the mediastinum or surrounding structures. As Veziant et al. mention in their work [1] from a pathophysiologic standpoint, esophageal rupture can arise from various factors, such as trauma, iatrogenic injury, or underlying esophageal pathologies like malignancies or strictures. Its etiology often involves a combination of factors, including forceful vomiting, foreign body ingestion, or iatrogenic causes like endoscopy. Clinically, patients may present with a spectrum of symptoms, ranging from severe chest pain and dysphagia to signs of sepsis or mediastinal emphysema as described by Kaul in his publication [2]. Timely diagnosis through imaging modalities like CT scans, contrast esophagography, or endoscopy is crucial for prompt management and favorable outcomes. 

Offering aggressive care in the setting of futile treatment for esophageal perforations raises several ethical, medical, and practical implications. If the prognosis is deemed futile due to factors such as extensive tissue damage, underlying comorbidities, or delayed presentation, aggressive care may only prolong suffering without meaningful improvement in outcomes. Opting for palliative measures in such cases focuses on enhancing the patient's quality of life and providing comfort rather than pursuing futile treatments. Palliative care can include pain management, nutritional support, and psychological support for both the patient and their loved ones. It also allows for discussions around end-of-life preferences, goals of care, and the opportunity to spend meaningful time with family and friends. It requires collaboration between the medical team, the patient, and their family, with a focus on shared decision-making and ensuring that the chosen approach aligns with the patient's goals and preferences. Ultimately, the implications of offering aggressive care versus palliative measures for esophageal perforations extend beyond medical considerations to encompass ethical, emotional, and practical dimensions, highlighting the importance of a holistic approach for each patient. 

Here, we present the case of a 66-year-old male with mid-esophageal perforation in the setting of an elongated esophageal mass due to metastatic squamous cell carcinoma. In this report, we will explore a case of esophageal rupture associated with malignancy and present an overview of the most common interventions for esophageal rupture. 

## Case presentation

The patient is a 66-year-old male with alcohol use disorder who presented with altered mental status. He was initially found on the floor of his room by his family member. The patient's last well-known was more than 24 hours prior to presentation. Per the family at the bedside, the patient was noted to be more withdrawn, with very minimal verbal interaction for the past month. Of note, the patient fell off his bike four months prior to presentation to the ED and sustained an injury to his chest but was never assessed due to the patient's refusal. 

On initial presentation, the patient was noted to be confused and disheveled, with minimally comprehensible speech. Physical examination was significant for an enlarged left supraclavicular lymph node; the patient was also significantly hypothermic (91.2°F) at the time. Initial labs showed significant leukocytosis with a left shift and severe electrolyte derangement, including severe hypercalcemia (18.8 mg/dL), hypernatremia (147 mEq/L), and severe hypokalemia (2.5 mEq/L). The urine drug screen was negative, and the patient's ethanol levels were negative. The patient met sepsis criteria on admission and was treated per sepsis protocol; he received IVF and was started on broad antibiotic coverage. The patient was admitted to the critical care unit for further management of metabolic encephalopathy with severe hypercalcemia and sepsis. The patient's code status on admission was full code. 

**Figure 1:**
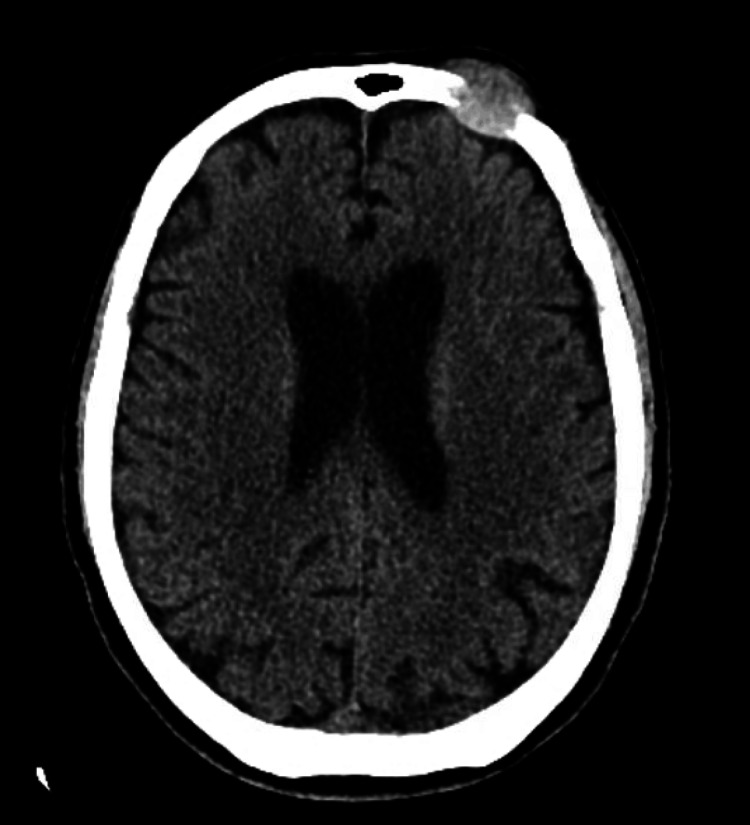
CT of the head without contrast. There is a 2.8x1.7x2.5 cm mass in the anterior left frontal skull with bony destruction, suggesting a neoplasm/metastasis. There is a mild mass effect on the anterior left frontal cortex due to the left frontal skull mass. There is no midline shift.

Due to the unknown source of sepsis and hypercalcemia, CT imaging of the head, chest, abdomen, and pelvis was performed. Non-contrast CT of the head identified a 2.8x1.7x2.5 cm mass in the anterior left frontal bone with bony destruction, likely neoplasm or metastasis (Figure 1). A mild mass effect to the anterior left frontal cortex was also noted.

**Figure 2:**
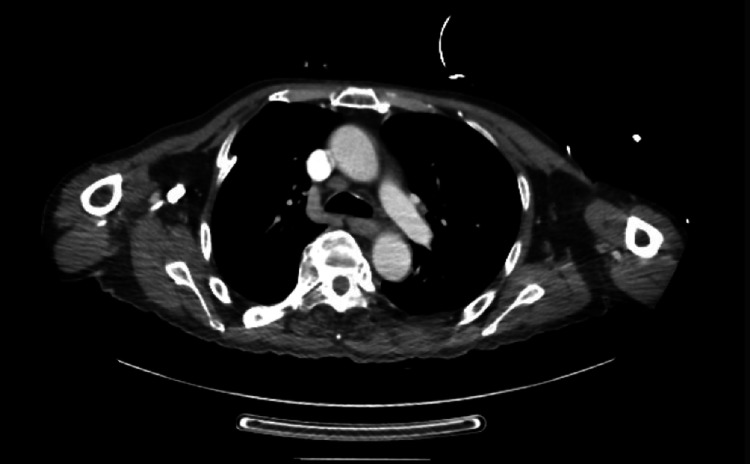
CT of the chest with contrast. Diffuse irregular wall thickening involving the mid- to distal esophagus suspicious for malignancy.

**Figure 3:**
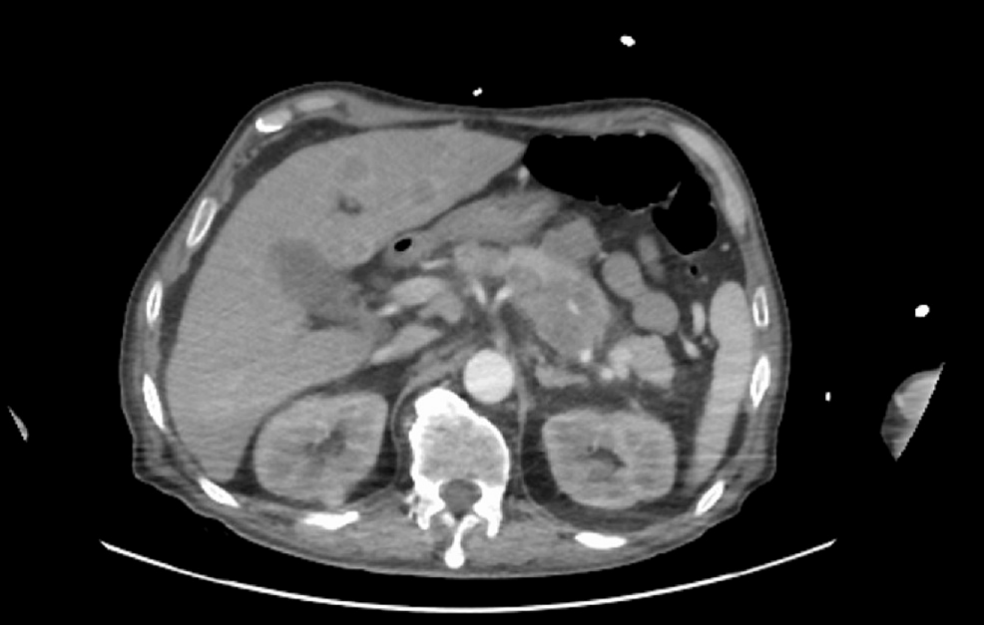
CT of the abdomen with contrast. Multiple hypodense ill-defined lesions throughout the liver compatible with metastatic disease. Extensive necrotic adenopathy involving the upper abdomen adjacent to the stomach and caudate lobe that measures at least 6x8.3 cm that extends into the body of the pancreas.

CT imaging of the chest, abdomen, and pelvis (Figure 2 and Figure 3) identified the following: diffuse irregular wall thickening of the mid- to distal esophagus extending into the stomach; lytic lesions of the left seventh rib, as well as L3, L5, and left iliac crest; multiple hypodense ill-defined lesions throughout the liver; extensive necrotic adenopathy of the upper abdomen adjacent to the stomach and caudate lobe of the liver, extending into the body of the pancreas; and bilateral supraclavicular and subcarinal adenopathy. These findings were all suspicious for metastases. 

**Figure 4:**
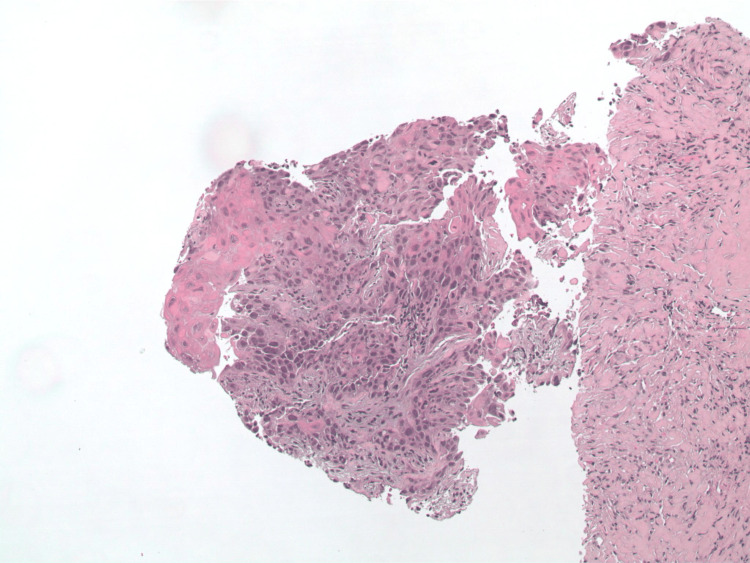
Left supraclavicular node lymph node core biopsy demonstrating metastatic squamous cell carcinoma.

The patient underwent a lymph node biopsy of the left supraclavicular lymph node, which demonstrated metastatic squamous cell carcinoma; see Figure 4 below for histopathology. Primary malignancy was suspected to be esophageal in origin given the cell type identified and location of metastases. 

**Figure 5:**
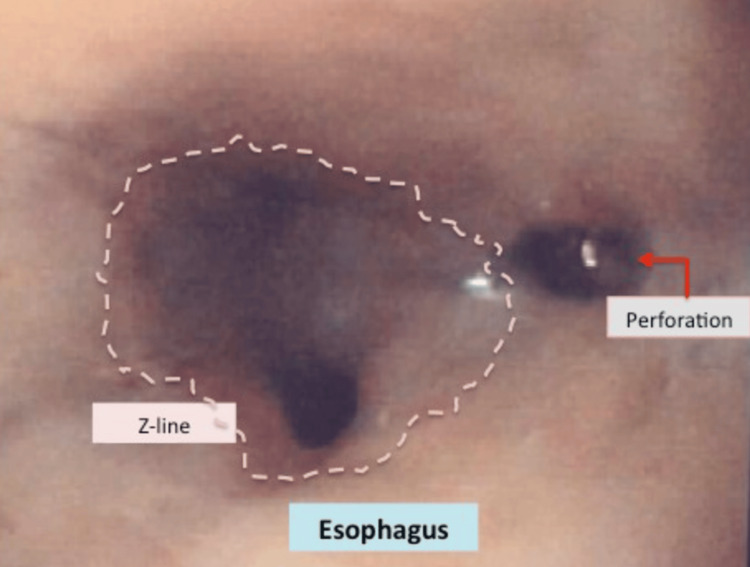
Esophageal perforation noted on endoscopy. Reference: [3]

Tumor markers CEA, PSA, CA 19-9, and CA 125 were negative; the PTH-related peptide was positive. Nephrology was consulted for the further management of electrolyte abnormalities; the patient received calcitonin for severe hypercalcemia. Hypercalcemia was thought to be in the setting of malignancy, given imaging findings. 

The patient's hospital course was complicated by an episode of aspiration pneumonia with multiple fever spikes. He was stabilized and gradually became more alert and oriented. On hospital day 9, the patient was noted to be hypertensive with SBP to 170s and tachycardic with labored breathing and audible wheezing. CT of the abdomen and pelvis and CT angiogram of the chest were performed; a new esophageal perforation (please see example perforation endoscopic image in Figure 5 above) along the region of the esophageal mass was identified with a small pneumomediastinum. 

The patient was evaluated by multiple specialties including hematology/oncology, surgical oncology, radiation oncology, cardiothoracic surgery, general surgery, gastroenterology, and palliative care; given the extent of metastatic disease and complications at this time, the patient was not deemed to be a good surgical candidate and was recommended for palliative care. 

Given this new finding of esophageal perforation, the patient was placed on nothing by mouth (NPO), and goals of care discussions were had with the patient and his family. The patient clearly expressed that he wanted everything done, with hopes of returning to his home country Mexico. The case was discussed with all involved specialties, and multiple attempts were made to transfer the patient to a tertiary care center with cardiothoracic or advanced gastroenterology services for evaluation and possible esophageal stenting for palliative feeds. 

The patient's code status was changed to do not resuscitate/do not intubate (DNR/DNI) after multiple discussions with the patient, but the patient still expressed a desire to undergo all interventions possible, including esophageal stenting. A Physician Orders for Life-Sustaining Treatment (POLST) form was filled out with the patient and his family, which clearly outlined the patient's wishes for all active treatment, including a short trial of total parenteral nutrition while awaiting transfer. The patient continued to express desires to eat and drink water. The patient's nutritional status continued to decline, and the patient became gradually more incapacitated, not being able to verbalize his current condition and prognosis as was previously explained to him. Psychiatry was consulted, and the patient was deemed to have the capacity to make decisions regarding feeding. The patient was eventually placed on comfort care to allow for comfort feeds and was placed on a morphine drip for pain associated with eating. The patient expired shortly after starting comfort feeds. 

## Discussion

For this 66-year-old male with a past medical history significant only for alcohol use disorder, his esophageal rupture in the setting of widespread metastatic disease ultimately led the patient and his family to opt for inpatient hospice. Evaluation by hematology, radiation oncology, and primary care team determined that the patient had a poor prognosis. Treatment options for this patient's mid-esophageal rupture were limited by the burden of underlying disease and futility of care. The standard of care for an esophageal rupture depends on the location and cause of the rupture. Options for initial management include esophageal stenting or surgical intervention. In an unstable patient, urgent stenting with devitalized tissue debridement and fluid drainage are indicated. While the patient remained hemodynamically stable, his underlying esophageal mass and widespread metastatic cancer meant he was not a candidate for thoracic surgery. As such, transfer to a tertiary care center was planned for evaluation for endoscopy with esophageal stent placement. The endoscopy in this case was planned as a palliative measure; the patient remained NPO during the initial evaluation, and the transition back to enteral nutrition was optimal for palliation and was desired per the patient's request. Unfortunately, the patient's clinical status deteriorated, and the patient and his family opted for comfort care measures only before an accepting bed was available at the tertiary facility. 

Esophageal perforation can be managed surgically with approaches such as esophagectomy or primary esophageal repair or via surgical drainage. As the review by Kaul [2] mentioned, although the technical success rate can be upwards of 90%, up to 40-50% of patients may experience pain or other stent-related complications. As the studies by Veziant et al. [1], Singh and Rizk [4], and Nassour and Fang [5] mentioned, the repair approach can range from open to minimally invasive. Despite a variety of approaches, esophageal perforation still carries a substantial morbidity and mortality rate, with 90-day mortality rates highest in those with a malignant etiology. From the information gathered by Veziant et al. [1] European data from 2012 to 2021, a surgical approach had the best outcomes, and high-volume centers (defined as centers with >8 esophageal perforation cases per year) had significantly fewer deaths of patients who experienced complications within 90 days after esophageal perforation. Esophageal stenting, while a more conservative treatment approach compared to surgery, has multiple risks, including stent migration or mal-placement as mentioned by reviews by Schmitz et al. [6], Brinster et al. [7], and Axtell et al. [8].

Ultimately, the decision to offer this patient transfer to a tertiary facility in order to undergo evaluation for esophageal stenting was more of a palliative measure; the patient could not be fed due to metastatic masses within the stomach, leading G-tube placement to be unsafe due to the high risk of erosion and peritonitis. Along with the large mid-esophageal rupture, the options for enteral feeding for this patient were limited. Oral feeding would soon lead to mediastinitis and death; parenteral nutrition was a mere temporizing measure. In the face of the patient's preexisting poor nutritional status due to metastatic cancer, dysphagia, and alcoholism before his hospitalization, the patient had minimal nutritional reserve. As the patient's mentation waned throughout the hospital course, the patient remained clear regarding one aspect of his care: he wanted to eat. Given the patient's strong desire to eat in the underlying setting of an esophageal perforation and untreatable metastatic cancer with a limited total parenteral nutrition trial, the dilemma of comfort care versus full treatment persisted. While clinically qualifying for hospice, the patient wanted to pursue full treatment options, including an esophageal stent that would allow him to eat and total parenteral nutrition as a temporizing nutrition alternative. However, in an effort to reach an end goal of eating, the patient experienced emotional distress from not being able to consume solids or liquids by mouth. This was evident by the patient opening ice packs while NPO and eating ice chips. 

Palliative measures such as esophageal stenting, as in the above case, can provide comfort to patients. Offering palliative stenting would have allowed him to eat and provided the patient comfort and joy in his final days.

## Conclusions

The management of esophageal perforation in patients with underlying metastatic disease is complex as treatment options are limited by the underlying comorbid illness and futility of care. Surgical interventions such as esophagectomy or primary esophageal repair have high rates of morbidity, mortality, and complications when due to malignant etiology. In the case of our patient, whose deteriorating clinical status precluded surgical intervention, offering palliative measures such as esophageal stenting emerged as a compassionate approach to alleviate discomfort and enable oral intake. 

## References

[1] Veziant J, Boudis F, Lenne X, Bruandet A, Eveno C, Nuytens F, Piessen G (2023). Outcomes associated with esophageal perforation management: results from a French nationwide population-based cohort study. Ann Surg.

[2] Kaul V (2023). Stenting for esophageal cancer. Gastroenterol Hepatol (N Y).

[3] Søreide JA, Viste A (2011). Esophageal perforation: diagnostic work-up and clinical decision-making in the first 24 hours. Scand J Trauma Resusc Emerg Med.

[4] Singh NP, Rizk JG (2008). Oesophageal perforation following ingestion of over-the-counter ibuprofen capsules. J Laryngol Otol.

[5] Nassour I, Fang SH (2015). Gastrointestinal perforation. JAMA Surg.

[6] Schmitz RJ, Sharma P, Badr AS, Qamar MT, Weston AP (2001). Incidence and management of esophageal stricture formation, ulcer bleeding, perforation, and massive hematoma formation from sclerotherapy versus band ligation. Am J Gastroenterol.

[7] Brinster CJ, Singhal S, Lee L, Marshall MB, Kaiser LR, Kucharczuk JC (2004). Evolving options in the management of esophageal perforation. Ann Thorac Surg.

[8] Axtell AL, Gaissert HA, Morse CR (2022). Management and outcomes of esophageal perforation. Dis Esophagus.

